# Strong Manual Acupuncture Manipulation Could Better Inhibit Spike Frequency of the Dorsal Horn Neurons in Rats with Acute Visceral Nociception

**DOI:** 10.1155/2015/675437

**Published:** 2015-07-15

**Authors:** Shouhai Hong, Shasha Ding, Fei Wu, Qiang Xi, Qiang Li, Yangyang Liu, Tao Zhou, Cai Qing, Yongming Guo, Yi Guo

**Affiliations:** ^1^Acupuncture Department, Zhejiang Provincial Hospital of TCM, No. 54, Youdian Road, Hangzhou 310006, China; ^2^Acupuncture, Moxibustion and Tuina College, Tianjin University of Traditional Chinese Medicine, No. 312, Anshan West Road, Nankai District, Tianjin 300193, China; ^3^Research Center of Experimental Acupuncture Science, Tianjin University of Traditional Chinese Medicine, No. 312, Anshan West Road, Nankai District, Tianjin 300193, China; ^4^Department of TCM, Wuhu First People's Hospital, No. 19, Jiehebeit Road, Jinghu District, Wuhu 241000, China; ^5^Department of Rehabilitation and Health Care, Hunan Traditional Chinese Medical College, Zhuzhou 412012, China

## Abstract

Afferent impulses from visceral nociception can be regulated by acupuncture at spinal cord level; however, the effects of different manual acupuncture (MA) manipulations on the afferent impulses are still unknown. Here, we analyzed the spike frequency of excitatory gastric-related wide dynamic range (WDR) neurons in spinal dorsal horn (SDH) following acute gastric distension (GD) in rats and compared their responses to MA manipulations with four different frequencies (0.5, 1, 2, and 3 Hz) at Zusanli (ST36). Results indicated that the spike frequency was increased by acute GD stimulation. Under acute GD circumstances, the spike frequency was further activated by weak MA stimulation (0.5 and 1 Hz), while being significantly inhibited by strong MA stimulation (2 and 3 Hz). After 10 minutes of the strong MA stimulation, same intensity of acute GD caused less spike frequency. Our previous researches had demonstrated that different MA manipulations could increase spike frequency in an intensity-dependent manner in normal rats; these findings suggest that acupuncture may have different modulatory effects depending on the state of the stomach. Since neuronal spike frequency was related to the level of nociception, the results suggest that strong MA manipulation may have better effect on acute visceral nociception.

## 1. Introduction

Acupuncture has been accepted to effectively treat pain diseases by inserting needles into the specific “acupuncture point” (acupoints), and the effect is manifested when the inserted needles are connected to an electrical current (electroacupuncture, EA) or manipulated by hand (manual acupuncture, MA) [1–3]. Manual acupuncture which involves the manipulation of the inserted needles by hand, such as lifting, thrusting, twisting, twirling, or other complex combinations, is more traditional and widely used in practice. Many Traditional Chinese Medicine (TCM) practitioners consider that this practice is closer to TCM theory because it allows individualized treatment for different syndromes of TCM diagnosis. Actually, MA manipulation is one of the key factors that induce the effects of acupuncture [4, 5]. Different MA manipulations, containing different stimulation parameters (including frequency, angle, and depth) [4, 6], could produce different physiological responses [7–11] and therapeutic effects [12–14]. However, the mechanism under these phenomenons remains unclear.

From a neurophysiological aspect, acupuncture is a somatosensory stimulation therapy, which is closely related to nervous system [15, 16]. Nervous system characterizes information in external stimulations by spatiotemporal encoding. When stimulated by different MA manipulations, nervous system can evoke different electrical signals, because the excited receptors and nerve fiber types are different [16, 17]. So we speculate that different MA manipulations can elicit different electrical signals of corresponding nervous system, further generating different clinical effects. Based on nonlinear dynamics analysis, our previous studies [16, 18] had confirmed that, in normal rats, spike discharges of both primary afferents in dorsal root ganglion and wide dynamic range (WDR) second-order neurons in dorsal horn evoked by different MA manipulations had distinguished chaotic features and different MA manipulations could increase the spike frequency in an intensity-dependent manner. This showed that different MA manipulations evoked various kinds of neural electrical signals. Those results indicated that we could separate different MA manipulations with the characteristic of these neural electrical signals. However in pathological state, the electrophysiological properties of electrical signals evoked by different MA manipulations are still unknown. Therefore in this study, we further analyzed the effects of different MA manipulations on electrical signals (spike frequency) of excitatory gastric-related neurons in SDH in rats with visceral nociceptive stimulation (induced by acute gastric distension).

## 2. Materials and Methods

### 2.1. Animals Preparations

Experiments were performed on 35 adult male Wistar rats (weight: 240–280 g), which were purchased from the Tianjin Mountains and Red Experimental Animal Science and Technology Co., Ltd. (Animal license number SCXK (Jin 2009-0001)). All manipulations and procedures were carried out in accordance with the Guide for Care and Use of Laboratory Animals issued by USA National Institutes of Health and were approved by the Animal Ethics Committee of Tianjin University of Traditional Chinese Medicine in China. Rats were housed (23 ± 1°C) in groups and maintained under a 12-hour light/dark cycle with food and water available ad libitum. After an overnight fast of 18 h, anesthesia was induced with urethane (1.5 mg/kg, i.p.) and subsequently maintained with urethane (0.3 mg/kg/h, i.p.). The anesthesia was maintained at a level where noxious pinch stimuli did not produce flexion reflexes. The trachea was cannulated to provide unobstructed ventilation. Saline with 5% dextrose (1 mL·h^−1^, i.p.) was administered after surgery. Core body temperature was maintained at about 37.5°C by a feedback-controlled heating pad.

### 2.2. The Model of Acute GD

The stomach was carefully intubated with flexible Tygon tubing (2.3 mm OD and 1.3 mm ID) via the mouth, esophagus, and cardia, and the damage to the esophagus was minimized. For acute GD, a latex balloon (length: 2.5 cm; diameter: 2 cm; maximum volume 30 mL) was attached to the front of the Tygon tubing. A syringe (20 mL) was connected to the other end of the catheter to inflate and deflate the balloon with air, while a pressure transducer (Powerlab 8/35, AD Instruments Co., Australia) through a T-connection was used to monitor balloon pressure continuously [19]. According to [20–22], we defined an acute GD when intragastric pressure was 40 mmHg (about 15 mL air, visceral nociceptive model). During whole experiment, the intragastric pressure was consistent. After completion of the experiment, the stomach was exposed to confirm the placement of the balloon. Only animals in which the balloon was observed to be within the stomach were used for data analysis.

### 2.3. Extracellular Recording

This method has been reported in our previous study [18]. Briefly, after exposing the lumbar enlargement (L3–L5 spinal segments) by laminectomy, rats were mounted in a stereotaxic head holder (SR-5R, Narishige, Japan) and stabilized with clamps attached to L2 and T12 vertebral processes. The dura was carefully removed, and the spinal cord was covered with warm mineral oil (37°C). Extracellular recordings of the activity of single WDR neurons in SDH with receptive fields (RFs) located on ST36 were obtained with a glass microelectrode (impedance: 10~15 MΩ; the tip of the microelectrode: 1-2 *μ*m (A-M Systems Co.)) filled with 2% Pontamine Sky Blue in 0.5 mol/L sodium acetate. Recording electrodes were lowered into the spinal cord using an electronic micromanipulator (MO-10, Narishige, Japan) in 1 *μ*m steps: 0.5–1.2 mm lateral to the midline in the right side of spinal cord and 0.3–1.3 mm from the dorsal surface [23]. Recordings were made only from single neurons whose amplitude could be easily discriminated. Electrophysiological activity was amplified (DAM50, WPI, USA), filtered (bandwidth: 300–5000), audiomonitored, and recorded with data acquisition systems (MP150, Biopack, CA, USA). The recording site and ST36 were all in the right side of the rats.

### 2.4. Functional Classification of Spinal Neurons

As reported in our previous study [18], mechanical stimulations, such as stroking the skin or mild pinching, were applied to the RFs. Innocuous stimulation included stroking or lightly pressing the skin with a cotton swab. Noxious stimulation covered mild pinching with the experimenter's fingers or serrated forceps, but the latter stimulation was applied sparingly to avoid neuronal sensitization. Neurons were classed functionally according to their responses evoked by mechanical stimulations as (1) low threshold (LT) if they were excited maximally by innocuous stimulation, (2) WDR if they responded in a graded manner to innocuous tactile stimuli, light pressure, and noxious pinch, and (3) high threshold (HT) if they only responded to noxious stimulation.

### 2.5. Defining Convergent WDR Neurons

Convergent WDR neurons means all those WDR neurons recorded in SDH could respond to the stimulations induced by GD and MA at ST36. In this experiment, only these WDR neurons were studied.

### 2.6. Acupuncture

An acupuncture needle (0.25 mm × 25 mm, Tianjin Hua Hong Medical Co., Ltd., Tianjin, China) was inserted vertically into the right ST36 (on the anterolateral side of the hind limb near the anterior crest of the tibia below the knee under the tibialis anterior muscle). After* de qi*, ST36 was stimulated with four kinds of lifting-inserting MA manipulations with different frequencies (Table 1) for 2 minutes in four different groups. In the control group, no MA manipulation was applied. The procedure was performed by the same licensed acupuncturist who used metronome to keep the rhythm. Before application of MA manipulations on rat, the manipulations were repeatedly practiced on the ATP-II acupuncture manipulation parameter tester (which was manufactured by Shanghai University of Traditional Chinese Medicine, ShangXin Medical Technology Company), to make sure that the angle, depth, and frequency of lifting-inserting manipulation were consistent and repeatable [11].

### 2.7. Experimental Procedure

**Figure 1:**
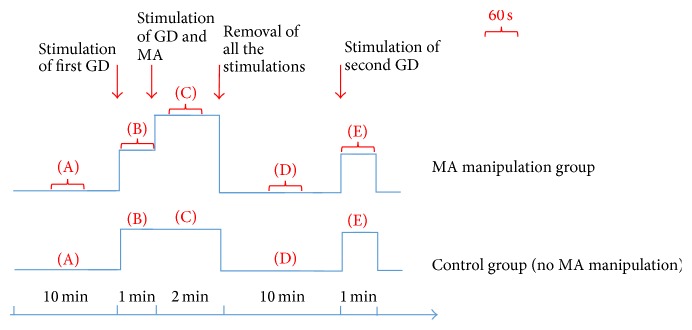
Outline of experimental protocol.

After finding the desired convergent WDR neurons in each rat, a 10-minute stabilization period was allowed. Then, a 10-minute baseline was recorded before the stimulation of GD. Then first acute GD was performed for 1 minute. Subsequently, 35 rats were randomly divided into 5 groups: control group (no MA manipulation; *n* = 7) and 4 different frequencies of MA manipulations groups (0.5 Hz group, 1 Hz group, 2 Hz group, and 3 Hz group, *n* = 7). MA manipulation was performed for 2 minutes and same intensity of acute GD (second GD) was given again after 10 minutes. The following measurement periods were analyzed: (A) 60 seconds before the stimulation of GD (baseline), (B) 60 seconds during GD stimulation, (C) 60 seconds of stimulation of GD and different MA manipulations on ST36, (D) 60 seconds after MA manipulations (second baseline), and (E) 60 seconds during second GD stimulation (Figure 1).

### 2.8. Data Preprocessing and Analysis

**Figure 2:**
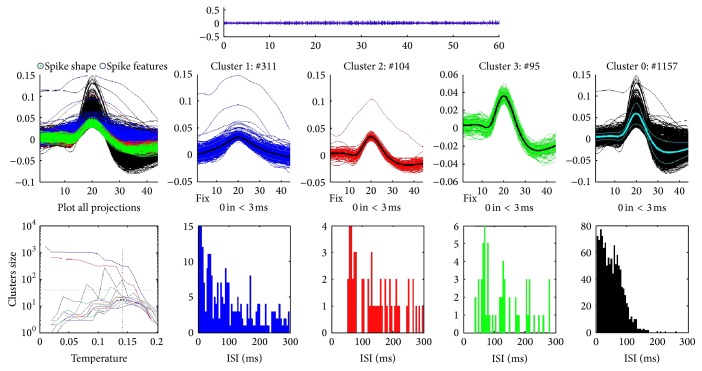
The spikes of neurons close by the electrode tip were separated via spike sorting. Here, three different components (marked in blue, red, and green) were distinguished.

As reported in our previous study [18], spike discharges of WDR neurons evoked by GD and different MA manipulations were recorded in sequence. The electrode may measure a different contribution from each of the different neurons around the microelectrode tip. Since the spike shape was unique and quite reproducible for each neuron, spike sorting algorithms using the shape(s) of waveforms were employed to distinguish the activity of one or more neurons from background electrical noise. As shown in Figure 2, three different components (marked in blue, red, and green) were distinguished.

Rate coding is a traditional coding scheme, in which information about the stimulus is contained in the firing rate of the neuron. In the present work, a time-window spike frequency analysis method was proposed to reflect the intensity changes of the discharges evoked by GD and MA at different frequencies [18].

Statistical comparisons were carried out with SPSS using one-way analysis of variance (ANOVA) and post hoc tests. All values were expressed as mean ± SD. For all comparisons, *P* < 0.05 was accepted as indicating significant differences.

## 3. Results

### 3.1. General Characteristics of WDR Neurons in SDH

**Figure 3:**
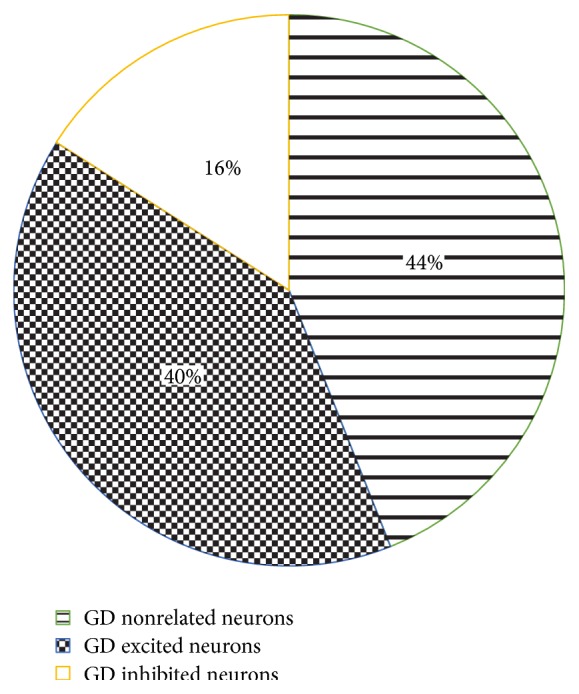
Different effects of GD on WDR neurons in SDH. 87 WDR neurons recorded in SDH. There were 38 GD nonrelated neurons (44%), 35 GD excited neurons (40%), and 14 GD inhibited neurons (16%).

**Figure 4:**
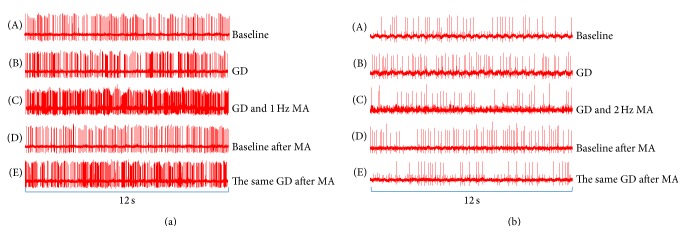
Examples of the extracellular activity of excitatory gastric-related WDR neurons in SDH. (a) The change in excitatory gastric-related WDR neurons caused by GD and (or) 1 Hz MA at ST36. (b) The change in excitatory gastric-related WDR neurons caused by GD and (or) 2 Hz MA at ST36.

Of all the 87 WDR neurons (most of the WDR neurons showed background activities in the absence of stimulation) recorded in SDH after gastric distention, 49 neurons were sensitized by the stimulation. Amongst them, 35 (40%) were excited and 14 (16%) were inhibited by GD (Figure 3). They showed more than 15% change (change rate = the number of spikes after stimulation/the number of spikes before stimulation) in the number of spikes and were defined as gastric-related neurons [23]. In the experiment, we focused on the excitatory gastric-related neurons. At the same time, those neurons were sensitized by MA at ST36. In Figure 4, examples of the extracellular activity of excitatory gastric-related WDR neurons in SDH were evoked by GD and (or) MA with different frequencies at ST36.

### 3.2. Effects of GD and Different MA Manipulations on Spike Frequency of Excitatory Gastric-Related WDR Neurons in SDH

**Figure 5:**
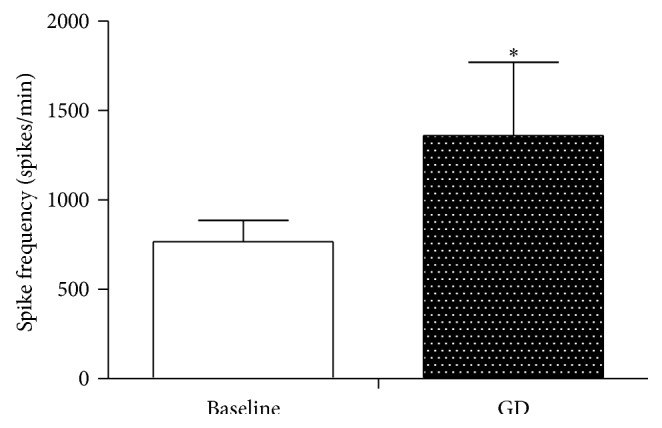
The activation effect of GD on excitatory gastric-related WDR neurons in SDH. After GD stimulation, the spike frequency of excitatory gastric-related WDR neurons in SDH was increased from 766 ± 119 to 1361 ± 408 spikes/min (^*∗*^ *P* < 0.05).

In the experiment, we examined 35 excitatory gastric-related WDR neurons on their reactions to GD stimulations. As showed in Figure 5, the degree of activation was significantly increased, with the spike frequency increased from 766 ± 119 to 1361.6 ± 408 spikes/min (*P* < 0.05). This indicated that visceral nociceptive stimulation could activate the activity of excitatory gastric-related WDR neurons in SDH.

**Figure 6:**
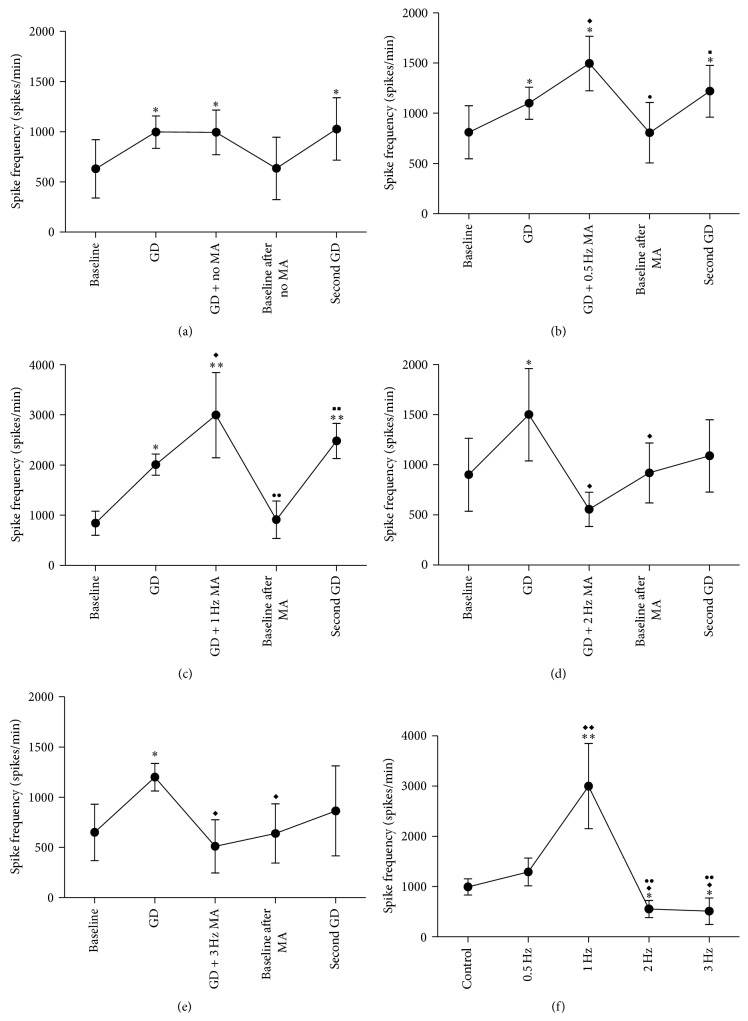
Spike frequency responses of excitatory gastric-related WDR neurons in SDH to GD and different MA stimulations. (a) Control group, without MA stimulation. (b) 0.5, (c) 1, (d) 2, and (e) 3 Hz MA stimulation, respectively. (f) Spike frequency changes evoked by different MA stimulations. In (a), (b), (c), (d), and (e), compared with baseline, ^*∗*^ *P* < 0.05 and ^*∗∗*^ *P* < 0.01; compared with GD, ^*◆*^ *P* < 0.05 and ^*◆◆*^ *P* < 0.01; compared with GD + MA, ^●^ *P* < 0.05 and ^●●^ *P* < 0.01; compared with baseline after MA, ^■^ *P* < 0.05 and ^■■^ *P* < 0.01. In (f), compared with control, ^*∗*^ *P* < 0.05 and ^*∗∗*^ *P* < 0.01; compared with 0.5 Hz, ^*◆*^ *P* < 0.05 and ^*◆◆*^ *P* < 0.01; compared with 1 Hz, ^●●^ *P* < 0.01.

In order to further explore the effect of different MA manipulation on neural electrical signals under visceral nociception, changes in spike frequency of excitatory gastric-related WDR neurons to 4 different frequencies (0.5 Hz, 1 Hz, 2 Hz, and 3 Hz) of MA manipulation at ST36 were observed under GD stimulation. Results showed that spike frequency was further excited by 0.5 Hz and 1 Hz MA stimulation, with an increase of firing rates from 1100 ± 160 to 1495 ± 271 spikes/min (*P* < 0.05; (b) in Figure 6) and from 2011 ± 160 to 2998 ± 848 spikes/min (*P* < 0.01; (c) in Figure 6), respectively. On the other hand, the activity significantly decreased for 2 Hz and 3 Hz MA stimulation. The spike frequency decreased from 1500 ± 460 to 555 ± 170 spikes/min (*P* < 0.05; (d) in Figure 6) and 1200 ± 167 to 512 ± 263 spikes/min (*P* < 0.05; (e) in Figure 6), respectively. However, in control group, spike frequency of excitatory gastric-related WDR neurons in SDH between GD and GD without MA manipulation stimulation was almost equal (*P* > 0.05; (a) in Figure 6). After MA manipulations (second baseline), the spike frequency returned to its original baseline in five groups. No significant difference of spike frequency was noted between two baselines (*P* > 0.05; Figure 6).

In addition, we compared the spike frequency of excitatory gastric-related WDR neurons among 4 different frequencies of MA manipulations. Compared with no MA and 0.5 Hz MA stimulation, the spike frequency significantly increased to 1 Hz MA stimulation (*P* < 0.01), while being significantly decreased to 2 and 3 Hz MA stimulation (*P* < 0.05; (f) in Figure 6). Compared with 1 Hz MA stimulation, the spike frequency also significantly decreased to 2 Hz and 3 Hz group (*P* < 0.01).

Next, in order to further investigate the effect of different frequencies of MA manipulations on GD, in the present research, same GD stimulation was given again after 10 minutes of different MA manipulations. We found, compared with the baseline, that the spike frequency of excitatory gastric-related WDR neurons evoked by the second GD was much more than the first GD before 0.5 and 1 Hz MA stimulation. The spike frequency was increased from 810 ± 263 to 1220 ± 257 spikes/min (*P* < 0.05; (b) in Figure 6) and 840 ± 243 to 2480 ± 348 spikes/min (*P* < 0.01; (c) in Figure 6), respectively. After 2 and 3 Hz MA stimulation, by contrast, the spike frequency evoked by the second GD was significantly decreased. Compared with the spike frequency of 900 ± 363 and 650 ± 281 spikes/min in baseline, the spike frequency decreased to 1088 ± 360 spikes/min (*P* > 0.05; (d) in Figure 6) and 864 ± 448 spikes/min (*P* > 0.05; (e) in Figure 6), respectively. However, in the control group (without MA manipulation), the spike frequency had little change from 997 ± 291.1 spikes/min at first GD and 1028 ± 310.5 spikes/min at second GD. Compared with the baseline (630 ± 291 spikes/min), they all had statistical difference (*P* < 0.05; (a) in Figure 6).

## 4. Discussion

ST36, innervated by deep peroneal nerve, is most frequently used to treat gastrointestinal diseases effectively [25, 26]. The spinal dorsal horn (SDH) is the first synaptic relay point for afferent pathways which plays an important role in modifying the transmission of noxious input [27]. Morphological study indicated that some neurons in the SDH could be commonly labeled by separate injection of neuroanatomical tracers into the stomach and ST36 in rats. Commonly labeled neurons were found in thoracic, lumbar, and sacral spinal segments [28]. Electrophysiological studies showed that noxious visceral and somatic afferent information separately from the gastrointestinal and ST36 could converge in the neurons of SDH [2, 23, 29, 30]. These findings revealed a potential mechanism for ST36 treating visceral diseases.

Pain, discomfort, and a sense of bloating in the upper abdomen were frequently reported by individuals suffering from functional dyspepsia and other functional gastric disorders. Studies have confirmed that sensory nerve fibers in vagus or splanchnic nerves that innervate the upper gastrointestinal tract provide information to the central nervous system, leading to varieties of consciously perceived sensations. Those sensations consist of satiety, nausea, bloating, discomfort, and pain, and the pain is associated with mechanoreceptor endings in muscle which respond to stretch or distension of the organ [31, 32]. Gastric distension (GD) model in rats have shown to be useful as a model of gastric mechano-nociception [21, 33, 34]. In this study, we observed that acute GD could increase the spike frequency of excitatory gastric-related WDR neurons in SDH of rats. And the spike frequency also could be regulated by MA at ST36. This conclusion is consistent with previous study [23].

Previous researches [23, 30] have confirmed that afferent impulses from visceral nociception could be regulated by acupuncture at spinal cord level; however, the effects of different MA manipulations are still unknown on the afferent impulses. From the classical theoretical and clinical perspectives of TCM, MA manipulation directly influences the effects of acupuncture. Given that MA manipulation is a kind of physical sensory stimulus, different MA manipulations can activate different kind of activated receptors or nerve fibers [2, 35]. Kagitani et al.'s study indicated that MA stimulation to ST36 activated afferent nerve fibers belonging to all four groups of afferents in rats. It was suggested that all four groups of somatic afferents activated by MA stimulation would elicit various effects when action potentials were delivered to central nervous system [36]. In fact, many human and animal studies had shown that different MA manipulations had different physiological responses [7–9, 9–11] and different therapeutic effects [12–14]. Therefore, we presumed that different MA manipulations have different effects on afferent impulses from visceral nociception.

In previous studies [12, 18], we have confirmed that, in normal rats, different MA manipulations could increase the spike frequency of both primary afferents in dorsal root ganglion and wide dynamic range (WDR) second-order neurons in dorsal horn in an intensity-dependent manner. In the present study, under the pathological condition of acute visceral nociception, we further analyzed the effect of MA manipulations with four different frequencies (0.5, 1, 2, and 3 Hz) at ST36 on spike frequency of excitatory gastric-related WDR neurons in SDH. We found that the increased spike frequency evoked by acute GD stimulation was significantly facilitated by weak MA stimulation (0.5 and 1 Hz), while being inhibited by strong MA stimulation (2 and 3 Hz), which meant different intensity of MA manipulations could result in opposite effect. These results are not consistent with our previous study [18]; there are two possible reasons. Firstly, the state of stomach was different. In previous study, normal rats were observed, while rats with acute visceral nociception induced by gastric distension were observed in present study. Like Lee et al.'s study [37], they investigated the interference of the brain activation during a passive movement task by retained acupuncture at ST36 and compared these effects between normal brain and Parkinson's disease brain. Results found that, depending on the brain pathologic conditions, different brain modulations effects were produced, even if acupuncture needles had been inserted at the same acupoint. Thus, we suggest acupuncture at ST36 would have different modulatory effect on spike frequency of excitatory gastric-related WDR neurons depending on the pathologic conditions of the stomach. Secondly, WDR neurons studied in the two studies were different. In our previous study, WDR neurons with receptive fields located on ST36 were studied, while, in the present study, only convergent WDR neurons were chosen. Which means all those WDR neurons with receptive fields located on stomach and ST36. Difference of the observed WDR neurons may lead to different results. In the future, we will further investigate the mechanisms of the differences.

Another part of our experiments showed that, compared to the first acute GD, after 10 minutes of MA stimulations with 2 and 3 Hz, second acute GD evoked less spike frequency of excitatory gastric-related WDR neurons. According to the pattern theory of pain [30], which assumes that neuronal discharge rate is related to the level of nociception, reduced discharge of these nociceptive neurons in response to GD may mean visceral pain is alleviated. So we speculate that strong MA stimulations (2 and 3 Hz) might have better effect on acute visceral nociception than weak stimulations. However, the effect indexes of acute gastric pain were not observed in our study. So what is the relation between the clinical effect and specific change of electrical signals? It has not been well clarified. In the future, we will further study the relationships among the different MA manipulations, electrical signals, and effects of acupuncture, so that we could better understand the characteristics, laws, and mechanisms of MA manipulation.

## 5. Conclusions

MA manipulation with different frequencies (0.5, 1, 2, and 3 Hz) at ST36 could change the spike frequency of excitatory gastric-related WDR neurons in SDH in rats with acute visceral nociceptive stimulation. The increased spike frequency evoked by acute GD stimulation was significantly inhabited by strong MA stimulations (2 and 3 Hz). And after 10 minutes of strong MA stimulation, same intensity of acute GD caused less spike frequency. Combined with our previous research, these findings suggested that MA with different frequencies at ST36 had different modulatory effects depending on the state of body (normal rat or rat with acute visceral nociception). Since neuronal spike frequency was related to the level of nociception, we speculated that strong MA stimulation might have better effect on acute visceral nociception than weak stimulation.

## Figures and Tables

**Table 1:** Parameter of four different MA manipulations.

MA manipulation	Frequency (Hz)	Operation of MA	Depth (mm)	Duration time (second)
Lifting-inserting manipulation	0.5 1 2 3	Neutral reinforcement and reduction	5–7	120

MA, manual acupuncture. Neutral reinforcement and reduction (see [24]), needle body is perpendicular to the skin, and the force and amplitude of displacement of lifting and inserting are uniform and the distance of lifting and inserting is 2-3 mm.
